# scTEM-seq: Single-cell analysis of transposable element methylation to link global epigenetic heterogeneity with transcriptional programs

**DOI:** 10.1038/s41598-022-09765-x

**Published:** 2022-04-06

**Authors:** Kooper V. Hunt, Sean M. Burnard, Ellise A. Roper, Danielle R. Bond, Matthew D. Dun, Nicole M. Verrills, Anoop K. Enjeti, Heather J. Lee

**Affiliations:** 1 School of Biomedical Sciences and Pharmacy, College of Health Medicine and Wellbeing, University of Newcastle, Callaghan, NSW Australia; 2 Medical Genetics, Level 3 West, Hunter Medical Research Institute, Lot 1 Kookaburra Circuit, New Lambton Heights, NSW 2305 Australia; 3 NSW Health Pathology North, New Lambton Heights, NSW Australia; 4 Calvary Mater Newcastle, Waratah, NSW Australia

**Keywords:** DNA methylation, Methylation analysis, Next-generation sequencing, Acute myeloid leukaemia, Cancer epigenetics, Mobile elements

## Abstract

Global changes in DNA methylation are observed in development and disease, and single-cell analyses are highlighting the heterogeneous regulation of these processes. However, technical challenges associated with single-cell analysis of DNA methylation limit these studies. We present single-cell transposable element methylation sequencing (scTEM-seq) for cost-effective estimation of average DNA methylation levels. By targeting high-copy SINE Alu elements, we achieve amplicon bisulphite sequencing with thousands of loci covered in each scTEM-seq library. Parallel transcriptome analysis is also performed to link global DNA methylation estimates with gene expression. We apply scTEM-seq to KG1a acute myeloid leukaemia (AML) cells, and primary AML cells. Our method reveals global DNA methylation heterogeneity induced by decitabine treatment of KG1a cells associated with altered expression of immune process genes. We also compare global DNA methylation estimates to expression of transposable elements and find a predominance of negative correlations. Finally, we observe co-ordinated upregulation of many transposable elements in a sub-set of decitabine treated cells. By linking global DNA methylation heterogeneity with transcription, scTEM-seq will refine our understanding of epigenetic regulation in cancer and beyond.

## Introduction

Single-cell analysis of DNA methylation has revealed epigenetic heterogeneity in development and disease, and parallel transcriptomic analyses are allowing this heterogeneity to be understood in the context of genomic regulation^1,2^. For example, single-cell analysis of DNA methylation, chromatin accessibility and gene expression has demonstrated that active epigenetic remodelling is required for endoderm and mesoderm specification during gastrulation^3^. In contrast, the ectoderm lineage is epigenetically primed in the epiblast and serves as a default differentiation pathway. Similar analyses have been applied to colorectal cancer revealing relationships between somatic copy number alterations, DNA methylation and gene expression^4^. While genetic sub-lineages were found to have distinct epigenetic profiles, comparison between primary and metastatic sites suggested that epigenetic reprogramming was not essential for tumour dissemination.

The studies described above demonstrate the power of linking DNA methylation heterogeneity with genetic and transcriptional heterogeneity. However, technical challenges continue to limit the implementation of single-cell DNA methylation analyses. Most methods rely on bisulphite conversion to distinguish methylated from unmodified cytosines. This chemistry provides single-nucleotide resolution but is incompatible with available high-throughput droplet barcoding technologies. Thus, single-cell analysis of DNA methylation is currently limited to low-throughput multi-well plate assays that are relatively high cost. Furthermore, genome-wide bisulphite sequencing (BS-seq) requires ten times as many reads as RNA sequencing (RNA-seq), meaning that studies on thousands of cells are usually cost-prohibitive. Finally, the sparse data obtained from single-cell BS-seq (scBS-seq) and single-cell RNA-seq (scRNA-seq) libraries poses a major challenge to multi-omic studies hoping to identify individual loci where DNA methylation correlates with gene expression. In the study of colorectal cancer mentioned above^4^, promoters with differential DNA methylation between primary tumour and metastatic sites were identified, but no correlations to expression of the associated genes were reported. Indeed, the most exciting findings from this study were related to global changes in DNA methylation, as opposed to locus-specific effects.

We reasoned that assessment of global DNA methylation in single cells would be a useful alternative to genome-wide analyses in contexts such as embryonic development and cancer, and reckoned that transposable element (TE) methylation could be exploited for this purpose. TEs are conserved DNA sequences capable of replicating and inserting into new positions in the genome. Discovered by Barbara McClintock in 1950^5^, TEs are estimated to make up around half of the human genome^6^. Poly-A retrotransposons Long Interspersed Element 1 (LINE-1) and Short Interspersed Element Alu (SINE Alu) account for almost 25% of the genome and are some of the only active or ‘hot’ TEs still capable of transposing in our genome^7,8^. Active retrotransposition causes genome instability, and because of this mutagenic potential, TEs are epigenetically silenced by high DNA methylation levels in internal promoters. Since TEs are so abundant in mammalian genomes, global changes in DNA methylation are correlated to changes in TE methylation in early embryonic development^9^, primordial germ cell development^10^, induced pluripotent stem cell (iPSC) reprogramming^11^ and cancer^12^. Indeed, even in the single-cell analysis of colorectal cancer discussed above, lineage-specific global DNA hypomethylation was associated with an over-representation of TE sequences (LTRs, LINEs)^4^.

These observations justify the use of TEs as surrogate measures for global DNA methylation levels, and LINE-1 and SINE Alu elements are common targets for bisulphite conversion-based analysis^13,14^. Here we adapt this approach for cost-effective analysis of global DNA methylation levels in a method called single-cell transposable element methylation sequencing (scTEM-seq). To achieve this, we perform targeted amplification of bisulphite converted SINE Alu and LINE-1 sequences. We apply scTEM-seq in acute myeloid leukaemia (AML) cells and detect global DNA methylation heterogeneity following treatment with the hypomethylating agent (HMA), decitabine (DAC). Parallel analysis of gene expression in the same single cells identifies links to immune processes, translation and induction of TE expression.

## Results

To investigate whether TEs might serve as surrogate measures for global DNA methylation levels in single-cell data, we first interrogated genome-wide scBS-seq data from a colorectal cancer patient (CRC01)^4^. We observed very strong correlations between DNA methylation within TE annotations and global methylation averages for both LINE-1 (*R*^2^ = 0.88, p < 2.2^–16^) and SINE Alu (*R*^2^ = 0.91, p < 2.2^–16^) families (Supplementary Fig. S1A). Furthermore, TE methylation was sufficient to identify sub-clonal differences in global DNA methylation (Supplementary Fig. S1B)^4^. This demonstrates that TE methylation in single-cell data can highlight biologically interesting heterogeneity in cancer cells.

**Figure 1:**
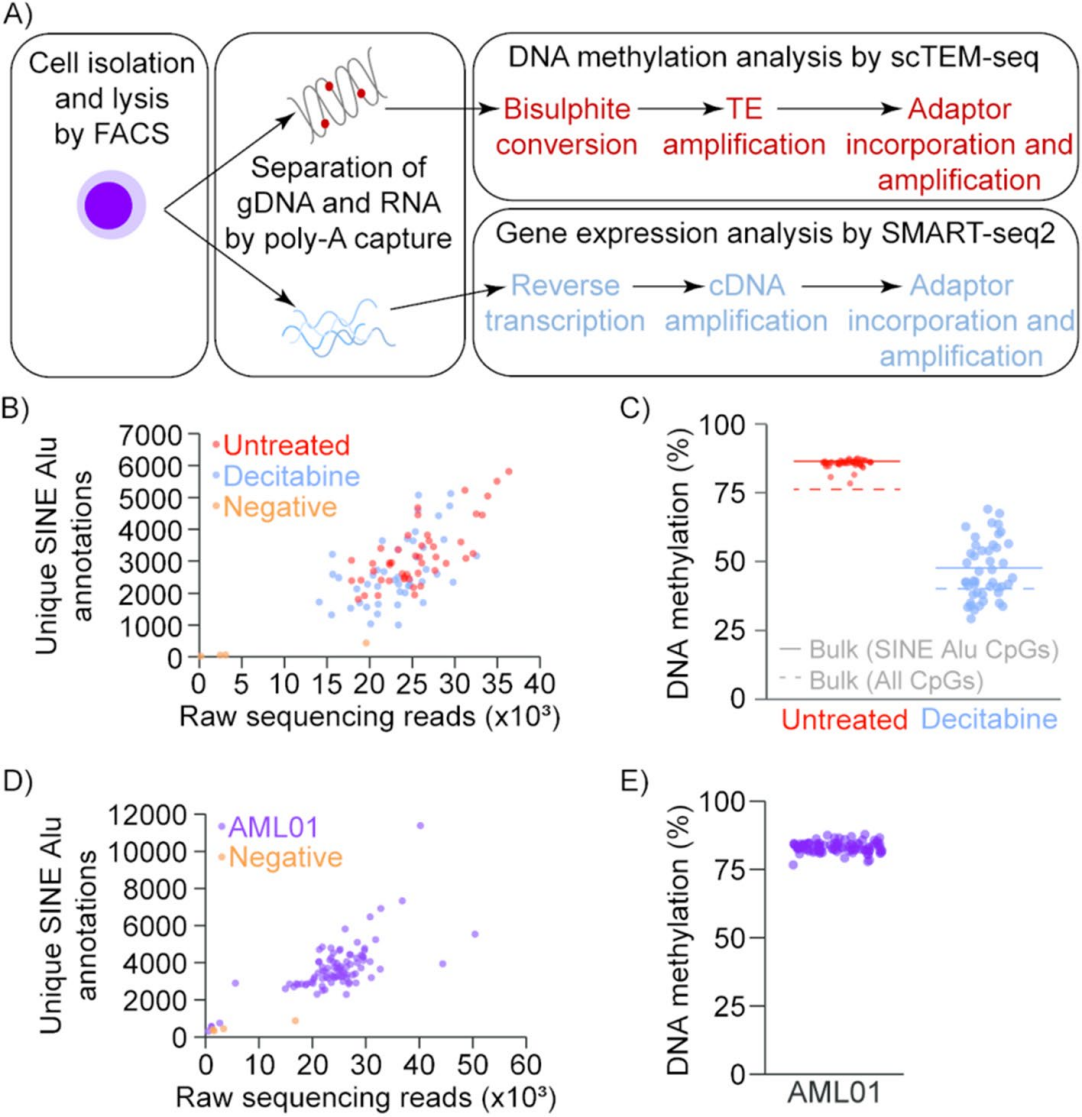
scTEM-seq accurately measures DNA methylation at TE sites. **(A)** Schematic representation of combined scTEM-seq and scRNA-seq workflow. **(B)** Unique SINE Alu sites measured in KG1a cells compared to raw sequencing reads. **(C)** DNA methylation levels as measured by scTEM-seq in KG1a cells with and without DAC treatment. Coloured lines show average DNA methylation levels at SINE Alu sites for each treatment group measured in bulk samples. DAC treated KG1a cells show a heterogeneous loss of DNA methylation. **(D)** Unique SINE Alu sites measured in AML01 patient blasts compared to raw sequencing reads. **(E)** DNA methylation levels in untreated AML01 patient blasts measured by scTEM-seq.

We adapted the scBS-seq protocol and achieved amplification of SINE Alu and LINE-1 sequences following bisulphite conversion of single-cell DNA samples (Fig. 1A, Supplementary Fig. S2A). LINE-1 primers used in previous studies^15^, and SINE Alu primers designed against an AluYa5 consensus sequence, were modified to be compatible with amplicon sequencing (see “Methods” and Supplementary Table S1). In initial experiments, SINE Alu primers (Sine.Alu_F, Sine.Alu_R) delivered greater amplicon yield and library complexity than LINE-1 primers (LINE.L1_F, LINE.L1_R), consistent with the higher copy-number of SINE Alu elements (Supplementary Fig. S2A). A second generation of 28 SINE Alu primer sequences were then designed (Supplementary Fig. S2, Supplementary Tables S1), and unique primer pairs were arranged in a 96-well plate (Supplementary Table S2). An 8 bp index was included in each primer, such that every library carried a dual index in the adaptor sequence, and a second internal dual index at the start of sequencing reads. This means that up to 18,432 scTEM-seq libraries can be pooled for sequencing. In addition, a 0–5 N spacer was included in these primers, and the direction of amplicon sequencing was reversed in 50% of primer pairs, to ameliorate the technical challenges of sequencing low-diversity amplicon libraries. For all further experiments, SINE Alu elements were targeted in scTEM-seq analysis.

We applied our optimised scTEM-seq protocol to Acute Myeloid Leukaemia (AML) cells treated with Decitabine (DAC); a hypomethylating agent (HMA) used to treat elderly AML patients^16^. DAC is a cytidine analogue that is incorporated into DNA and causes genome-wide loss of DNA methylation by blocking its maintenance during DNA replication. While some studies have demonstrated durable responses in some patients, clinical use of this epigenetic therapy is limited by variability in patient response^16^.

KG1a AML cells were treated with and without 100 nM DAC for 72 h, and single cells were collected by FACS for scTEM-seq. Negative controls (no cell) were also included to monitor DNA contamination in reagents. Average amplicon yield from single cell samples was 16.10 ng/μl (4.08 SD (standard deviation)), compared to 1.12 ng/μl (1.03 SD) in negative controls (Supplementary Fig. S3A). scTEM-seq libraries achieved unique alignment rates of 67.23% (5.11 SD) (Supplementary Fig. S3C), and efficient bisulphite conversion was confirmed by very low non-CpG methylation rates (DNA methylation in CHG trinucleotide contexts was 0.67%, 0.2 SD) (Supplementary Table S3). Information was recovered from between 1000 and 6000 unique SINE Alu annotations for each cell, despite low sequencing depth (14,000–37,000 raw reads per cell) (Fig. 1B). Further analysis confirmed that scTEM-seq reads were predominantly focused on AluY elements, though other SINE Alu families were also represented in the data (Supplementary Fig. S4).

In untreated KG1a cells, scTEM-seq showed uniformly high levels of DNA methylation, with an average of 85.4% (1.65 SD). In DAC treated cells, a heterogeneous loss of DNA methylation was observed, with levels ranging from 29 to 69% (average 41.86%, 10.46 SD) (Fig. 1C). To assess the accuracy of these DNA methylation measurements, we first compared our scTEM-seq results to genome-wide methylation levels in bulk sequencing libraries prepared from matched populations of cells. The average methylation rate for all CpGs covered in bulk libraries was 78.58% for untreated cells and 43.87% for DAC treated cells. As expected, CpGs within SINE Alu sites had higher average methylation levels at 86.48% and 47.63% in untreated and DAC treated cells, respectively (Fig. 1C). For untreated cells, 42 of 46 scTEM-seq libraries had methylation estimates within ± 2% of the expected value based on bulk libraries (86.48%). Thus, SINE Alu analysis by scTEM-seq provides accurate DNA methylation estimates.

To validate our observation of DAC induced DNA methylation heterogeneity, we compared scTEM-seq to an established method. The range and variance of DNA methylation values were similar for genome-wide single-cell bisulphite sequencing (scBS-seq)^17^ and scTEM-seq libraries (Supplementary Fig. S5A). Furthermore, scTEM-seq analysis of HL60 cells treated with and without DAC showed similar patterns of DNA methylation to KG1a cells (Supplementary Fig. S5B).

We also performed a bootstrapping analysis of scTEM-seq data to test the stability of DNA methylation estimates at low read counts. A slight bias toward increased methylation estimates at low read count was observed, especially in cells with high methylation rates (Supplementary Fig. S5). This is likely to result from more efficient amplification of methylated sequences, which is known to influence bisulphite sequencing libraries^18^. Nonetheless, methylation estimates were stable within a 3% range for all sub-samples of > 5000 aligned reads, demonstrating that scTEM-seq is a reliable measure of DNA methylation.

To test scTEM-seq in primary human cells, we applied our analysis on sorted blasts from an AML patient. Amplicon yield, alignment rates and bisulphite conversion were comparable to KG1a samples, and 88 of 92 libraries passed quality control with representation of > 1000 unique SINE Alu elements (Supplementary Table S4). This patient had not received hypomethylating agent therapy, and DNA methylation at SINE Alu elements was consistently high in these cells (84.74%, 2.15 SD) (Fig. 1E).

**Figure 2:**
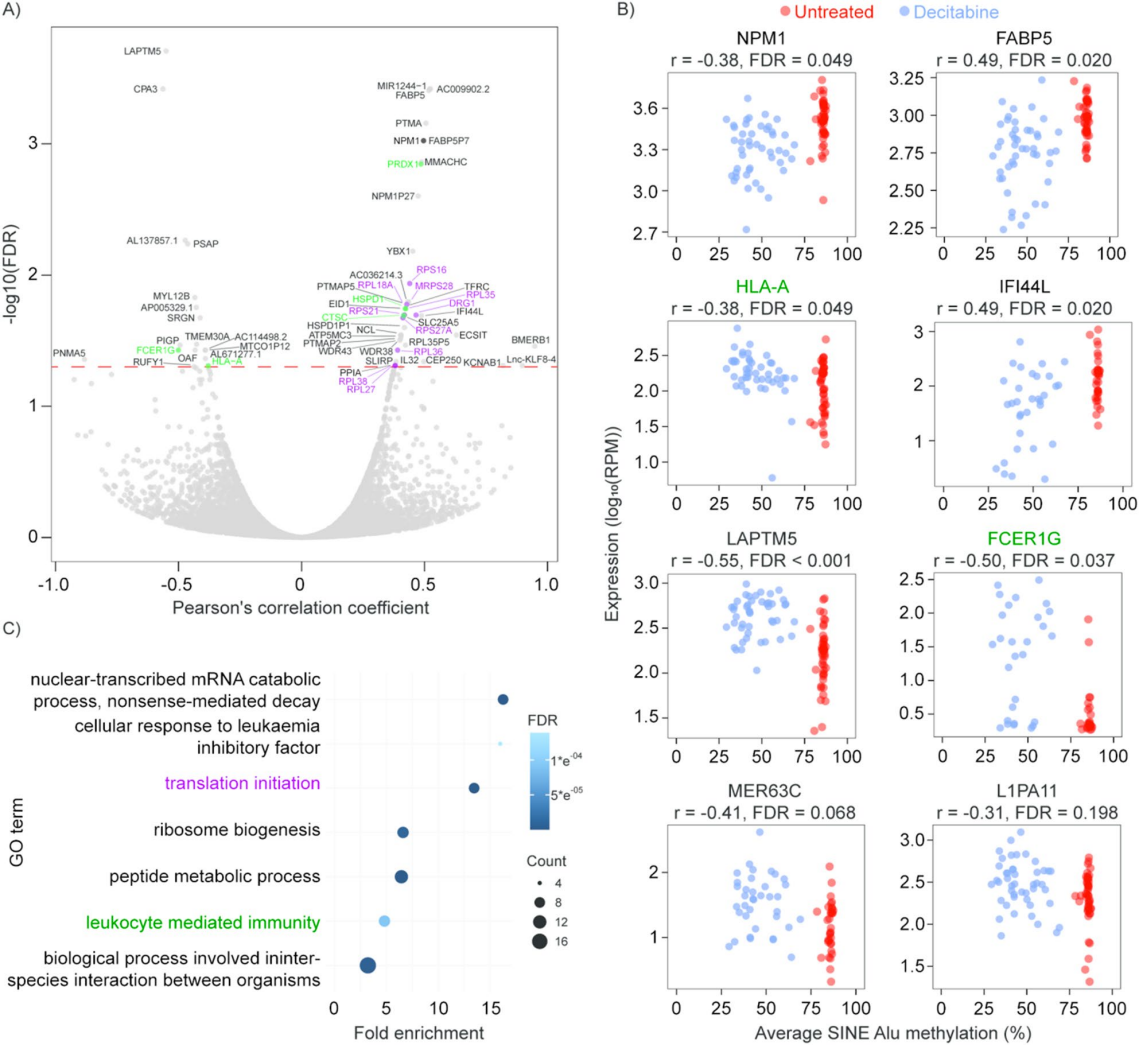
Correlations between average DNA methylation levels and gene expression. **(A)** Volcano plot showing Pearson’s correlation between average DNA methylation in SINE Alu elements and gene expression in the KG1a dataset. Genes involved in ‘translational initiation’ and ‘leukocyte mediated immunity’ are highlighted in purple and green, respectively. **(B)** Select examples showing expression levels of an individual gene and average DNA methylation levels in our treated and untreated KG1a cells. Examples include 6 genes (*NPM1*, *FABP5*, *HLA-A*, *IFI44L*, *LAPTM5* and *FCER1G*) and 2 TEs (MER63C and L1PA11). The Pearson’s correlation coefficient (r) and false discovery rate (FDR) for each correlation are shown. RPM = reads per million. **(C)** Gene ontology (Panther) results for statistically overrepresented biological pathways in all genes with expression correlated to DNA methylation (FDR < 0.05). For related terms, only the pathway with the highest number of correlated genes is displayed for simplicity.

Prior separation of gDNA and RNA allowed us to prepare matched scRNA-seq libraries from each cell using the SMART-seq2 protocol^17,19^ (Fig. 1A, Supplementary Tables S5 and S6). We then correlated DNA methylation levels to changes in gene expression. In our KG1a data, expression of 60 genes was correlated to the average DNA methylation at SINE Alu sites (FDR < 0.05 after multiple testing correction), with the majority (43) showing positive associations (Fig. 2A). For example, interferon induced protein *IFI44L* was down-regulated in cells with lower SINE Alu methylation, whereas major histocompatibility complex (MHC) I component *HLA-A* was up-regulated in cells with lower SINE Alu methylation (Fig. 2B). Gene ontology analysis on all genes with a significant correlation to DNA methylation (FDR < 0.05) revealed over-represented of pathways including: translational initiation, leukocyte mediated immunity, and biological process involved in interspecies interaction between organisms (Fig. 2C). These results are consistent with the ability of HMAs to induce differentiation^20^, and inhibit translation^21^, in AML cells.

**Figure 3:**
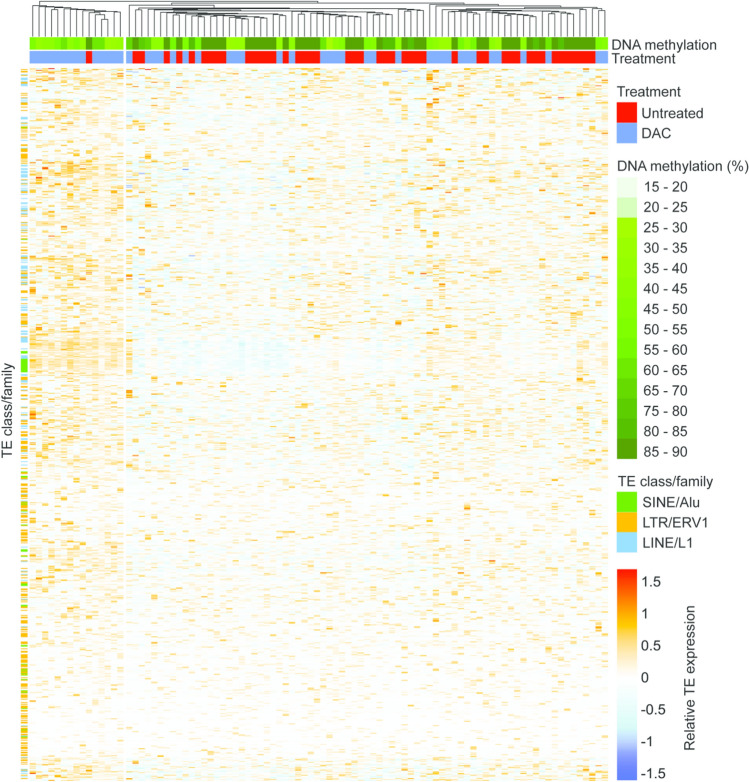
Coordinated up-regulation of TE transcription is observed in a subset of DAC treated KG1a cells. TE families with altered expression between untreated (red) and DAC treated (blue) KG1a cells were identified by differential expression analysis using DEseq2. The heatmap shows the relative expression of all TEs from significant families (adjusted p < 0.05) following normalisation by variance stabilisation transformation (vst) (DESeq2) and mean centering. Both rows (TEs) and columns (cells) are clustered by Euclidean distance. Global DNA methylation percentages for each cell are indicated (green scale at top) and selected TE families are highlighted (left). In total, 11 TE families reached the significance threshold (Family:Class; acro:Satellite, ERV1:LTR, ERVK:LTR, L1:LINE, Alu:SINE, ERVL:LTR, ERVL-MaLR:LTR, TcMar-Tigger:DNA, hAT-Charlie:DNA, MIR:SINE, L2:LINE), corresponding to 834 TE elements. A sub-cluster of mostly DAC treated cells (left) have high expression of TEs.

HMAs have also been shown to act through a ‘viral mimicry’ process whereby loss of DNA methylation induces transcription of TEs (e.g. endogenous retroviruses, LINEs and SINEs) and a subsequent type 1 interferon response in effected cells^22–24^. To test whether scTEM-seq could link epigenetic heterogeneity induced by DAC to expression of TEs, we assessed the abundance of TE sequences in RNA-seq data. KG1a cells showed a clear increase in TE expression levels after DAC treatment (Supplementary Figure S6A), and a bias toward negative correlations between TE expression and DNA methylation (although no TEs had significant correlations after multiple testing correction, see examples in Fig. 2B). A trend toward negative correlations was also observed in AML01 and HL60 datasets, with 4 TE families showing significant (FDR < 0.05) correlations in HL60 cells (Supplementary Fig. S6B). To further investigate TE expression patterns, we performed clustering analysis of TE families that were differentially expressed after DAC treatment (Fig. 3, Supplementary Figs. S7 and S8). In KG1a cells, we observed a subgroup of mostly DAC treated cells with co-ordinated up-regulation of many TEs, especially LINE-1 and SINE Alu families (Fig. 3). A similar pattern was observed in HL60 cells (Supplementary Fig. S8). Interestingly, cells with high TE expression could not be distinguished from other DAC treated cells based on global DNA methylation alone (KG1a: 46.4%, 10.9 SD vs 46.8%, 10.4 SD , respectively) (Supplementary Fig. S9), suggesting that other factors must regulate TE expression in the absence of DNA methylation.

## Discussion

TEs have been widely targeted for surrogate measures of global DNA methylation. We have adapted this approach to single cells, developing a cost-effective alternative to genome-wide techniques^17,25–29^. While other studies have amplified loci of interest in bisulphite converted DNA from single cells^30–34^, ours is the first to target TEs.

We demonstrate that methylation of SINE Alu elements in single cells compares well to global DNA methylation levels using in silico analysis of published data (Supplementary Fig. S1A) and by comparing scTEM-seq to matched bulk sequencing libraries (Fig. 2C), and established single-cell techniques (Supplementary Fig. S5A). SINE Alu methylation over-estimates global DNA methylation levels (e.g. by 6.8% for untreated KG1a cells) (Fig. 1C), which can be explained by the well-characterised enrichment of TEs in hypermethylated regions^35^. However, scTEM-seq accurately estimates SINE Alu methylation in untreated KG1a cells and detects changes in DNA methylation after DAC treatment (Fig. 1C). In untreated KG1a cells, scTEM-seq libraries had homogeneous SINE Alu methylation rates that were typically within ± 2% of the expected value from bulk measurements in a matched population of cells. Furthermore, down-sampling scTEM-seq libraries revealed that methylation estimates are stable, even at low read depth, for both treated and untreated cells (Supplementary Fig. S5C). scTEM-seq has several advantages over comparable genome-wide methods such as single-cell bisulphite sequencing (scBS-seq; Supplementary Table S6)^25^. scTEM-seq libraries are prepared using sequence-specific primers, rather than random-priming oligos, leading to reduced oligo contamination and improved alignment rates. Indeed, the unique alignment rates for scTEM-seq libraries are surprisingly high considering that repetitive loci are inherently difficult to map in the reference genome. Improved alignment rates confer a cost saving by reducing wastage from sequencing runs; however, an even greater advantage is obtained by reducing the sequencing demand. Whereas scBS-seq libraries require ~ 20 million raw reads per cell to obtain genome-wide information, scTEM-seq libraries can provide a global estimate of DNA methylation from ~ 20 thousand raw reads. Thus, the sequencing cost is 3 orders of magnitude lower for scTEM-seq libraries. Obviously, this reduced cost comes with a considerable loss of information. However, locus-specific analysis of DNA methylation is also difficult in genome-wide libraries, due to the low coverage obtained in each cell (e.g. 10–40% of the genome). Like scBS-seq and other plate-based methods, scTEM-seq is compatible with parallel analysis of gene expression in the same single cell. This allows epigenetic heterogeneity to be linked to transcriptional output. Thus, scTEM-seq will increase the scale of single-cell studies in biological contexts where global changes in DNA methylation are of interest.

In this study, DAC treatment of KG1a AML cells led to heterogeneous loss of DNA methylation and altered expression of many genes (Fig. 2). For example, *HLA-A* and *FCER1G* were negatively correlated to DNA methylation only 72 h after initial treatment, possibly signifying increased monocytic differentiation in cells that have lost DNA methylation^20^. Furthermore, we were able to link epigenetic heterogeneity to expression of TEs, suggesting that variable activation of viral mimicry pathways could influence treatment response. We identified a subgroup of DAC treated KG1a cells with co-ordinated up-regulation of many TE families. This group of cells could not be distinguished based on DNA methylation levels alone, suggesting that loss of methylation is insufficient for activation of viral mimicry. In cells that do not up-regulate TEs, other epigenetic processes may substitute for the suppressive effects of DNA methylation, or transcriptional activators required for TE expression may be absent. Interestingly, recent reports have implicated the histone methyltransferase SETDB1 in suppression of TEs and tumour immunogenicity, including effects in hypomethylated cell line models^36,37^.

A major limitation for the clinical use of hypomethylating agents is the variability in patient response. Although azacitidine has been shown to improve survival compared to conventional care, a large proportion of patients receive little or no benefit^38^. Changes in global DNA methylation levels during treatment, measured in bulk tumour samples, have not been able to predict patient response to hypomethylating agents^39,40^. Expression of subsets of evolutionarily young TEs, however, correlates with improved prognosis^23^. Using scTEM-seq, we can take these studies a step further and explore how heterogeneity of DNA methylation and expression of TE subtypes within a tumour contribute to patient prognosis.

We also applied scTEM-seq to primary patient blasts, revealing homogeneous levels of DNA methylation. We did not identify correlations between DNA methylation levels and gene expression in this small set of cells (data not shown). However, we did note a bias toward increased TE expression in cells with lower DNA methylation levels (Supplementary Fig. S6B). This is consistent with previous observations that DNA methylation proximal to TE sites correlates with their expression across different cancer types^41^. Future studies will apply scTEM-seq to many cells from numerous patients to test whether variation in TE methylation may lead to intra-tumoural heterogeneity in TE expression.

scTEM-seq is also relevant to several contexts in stem cell and developmental biology. iPSC reprogramming is a heterogeneous process in which global epigenetic remodelling accompanies reactivation of pluripotency networks^42,43^. Variable DNA methylation in iPSCs raises concerns regarding their safety in clinical regenerative medicine since incorrect reprogramming could lead to cancerous growth^44^. Thus, scTEM-seq may be a useful tool to understand the heterogeneity and assess the quality of iPSCs. Ultimately, scTEM-seq will find applications in many aspects of medicine and biology. The reduced complexity and cost of this approach will also allow multi-dimensional single-cell analysis to be used more often and at scale.

## Methods

### Cell lines and patient samples

KG1a cells (ATCC, catalog #CCL-246.1) were cultured in Iscove’s Modified Dulbecco’s Medium (IMDM) (Sigma-Aldrich, catalog # I3390) with 10% fetal bovine serum (FBS). HL60 cells (ATCC, catalog #CCL-240) were cultured in Iscove’s Modified Dulbecco’s Medium (IMDM) (Sigma-Aldrich, catalog # I3390) with 10% fetal bovine serum (FBS) and 4 mM glutamax (Life Technologies, catalog # 35050061). Routine mycoplasma testing was performed using the MycoAlert Mycoplasma Detection Kit (Lonza, catalog #LT07-318), and cell line validation was performed by the Australian Genome Research Facility using custom microsatellite analysis. Cell lines were treated with 100 nM 5-aza-2’-deoxycitidine (decitabine, DAC) every 24 h (0, 24 and 48 h) and harvested at 72 h.

Experiments involving human samples were approved by the human ethics committees of the Hunter New England Area Health service, and the University of Newcastle, and all methods were performed in accordance with the relevant guidelines and regulations. The AML patient included in this study (AML01) was recruited at diagnosis through the Calvary Mater Newcastle Hospital, with written informed consent. The patient was a 60-year-old male, diagnosed with secondary AML following chronic myelomonocytic leukaemia. Clinical assessment revealed a complex karyotype including an isochromosome 17q, and mutations in the *ASXL1*, *SETBP1* and *SRSF2* genes. Enriched mononuclear cells were purified from peripheral blood using Lymphoprep density gradient medium (StemCell, catalog # 7851) and SepMate tubes (StemCell, catalog # 85450), and cryopreserved.

### Cell sorting

KG1a cells were stained using the PE Annexin V Apoptosis Detection Kit (BD Life Science, catalog # 559763). Live cells (Annexin V^-^/7-AAD^-^) were sorted into individual wells of a 96 well plate containing lysis buffer 2.5μL RLT Plus Lysis Buffer (QIAGEN, catalog # 1053393) with 1U/μL SUPERase-In (ThermoFisher, catalog # AM2696). Before sorting, bulk KG1a samples of 1,000,000 cells were collected from both the untreated and treated populations for comparison with single cells. HL60 cells were stained with Propinium Iodide (PI) (ThermoFisher, catalog # P1304MP) and live cells (PI^-^) were sorted into 96 well plate containing lysis buffer 2.5μL RLT Plus Lysis Buffer with 1U/μL SUPERase-In.

Cryopreserved primary human cells were resuspended in thawing media (IMDM, 20% FBS), washed twice and resuspended. The cells were then rested for 1 h at 37 °C before preparation for flow cytometry. Cells (1 × 10^6^/100 μl) were stained with 1.5 μg/mL propidium iodide (PI, Sigma-Aldrich, P1304MP), 1:20 CD45-PECy7 (2D1, Life Technologies, catalog # 25-9459-42), 1:20 CD33-FITC (WM-53, Life Technologies, catalog # 11-0338-42) and 1:20 CD19-BV711 (SJ25C1, BD Biosciences, catalog # 563036). Single blasts (PI^−^/CD45^dim^) were collected in 2.5μL RLT Plus Lysis Buffer containing 1U/μL SUPERase-In in 96 well plates.

### Library preparation

We utilised the G&T-seq protocol to separate genomic DNA and RNA from the single-cell samples^45^. Genomic DNA from each cell was purified and bisulphite conversion was performed as described^17^, with minor modifications. Bisulphite conversion was carried out using the EZ-96 DNA Methylation-Direct MagPrep Kit (Integrated Sciences, catalog # D5054) with half volumes of the manufacturer’s instructions. Bisulphite converted DNA was eluted directly from MagBeads into PCR-mix, and amplification of TEs was performed with MagBeads still in the well. PCR cycling conditions used were 95 °C for 5 min (1 cycle), 98 °C for 20 s, 53 °C for 15 s, 72 °C for 1 min (35 cycles), and 72 °C for 10 min (1 cycle). PCR mix used 7.5 µl 1 × KAPA HiFi hotStart Uracil + ReadyMix (Millennium, catalog # ROC-07959079001) and 0.3 µM primer mix. Primers were targeted to SINE Alu and LINE-1 consensus sequences and included a partial adaptor sequence at the 5’ end to enable later indexing with NEBNext dual index oligos (Supplementary Fig. S2A, Supplementary Tables S1 and S2). Second generation primers also included a spacer of 0–5 N, and an 8 bp index sequence between the adaptor and SINE Alu priming sequence. After amplification libraries were purified using a 1.2 × volume of AMPure XP beads (Beckman Coulter, catalog # A63881). All libraries were then quantified using the Qubit dsDNA HS kit (Life Technologies), normalised and pooled to a single tube. Pools were then added to 0.8 µM NEBNext dual index oligos (Genesearch, catalog # E7780S) and 14.5 µl 1 × KAPA HiFi HotStart ReadyMix (Millennium, catalog # ROC-07958935001) for indexing and adaptor addition. PCR cycling conditions used were 98 °C for 45 s (1 cycle), 98 °C for 15 s, 65 °C for 30 s, 72 °C for 30 s (5 cycles), and 72 °C for 5 min (1 cycle). Pools were then purified using 0.9 × volume of Ampure XP beads, normalised and combined for sequencing. Matched scRNA-seq libraries were prepared as described^9,17^. For AML01, 4 columns (30 samples and 2 negative controls) were excluded prior to sequencing due to low library quality after an error in library preparation.

A post-bisulphite adaptor tagging (PBAT) approach^46^ was used to prepare bulk genome-wide sequencing libraries from matched populations of cells. Libraries were prepared as described^47^, with minor modifications. The 6NR adaptor 2 oligo used during second strand synthesis was modified (5’-CAGACGTGTGCTCTTCCGATCTNNNNNN-3’) to be compatible with NEBNext dual index oligos that were used for library amplification.

### Sequencing

Sequencing of bisulphite reads was performed using the Illumina MiSeq platform. Low read depth is required, so for data in this paper sequencing kits with only 4 million reads were used for 192 cells. Library loading concentrations of 8-10 pM were used with a 1% PhiX spike-in. We achieved on average 23,000 read pairs per sample.

scRNA-seq Libraries were sequenced using the NextSeq platform with a loading concentration of 1.5 pM and a 1% PhiX spike-in. We excluded all cells with alignment rates under 80%. With approximately 1,000,000 reads per cell, we measured between 6300 and 15,000 genes in all of our single cell KG1a scRNA-seq libraries (Supplementary Table S5). Gene numbers measured in AML01 cells were more modest, with between 2800 and 5200 genes in cells passing quality control (Supplementary Table S6).

PBAT libraries were sequenced using the MiSeq platform. These libraries were prepared with the intention of measuring global DNA methylation levels and as such were also sequenced with low read depth (~ 100,000 reads per bulk sample).

### Data processing and analysis (scTEM-seq)

After initial demultiplexing of primary Illumina indexes, Cutadapt (v2.10)^48^ was used to demultiplex pools based on custom secondary indexes (Supplementary Table S1). Commands –g and -G were used to pass named forward and reverse index lists as a .fasta file to Cutadapt. Bisulphite reads were trimmed using Trim Galore (v0.6.5)^49^. 10 bp was trimmed from both the 5’ and 3’ ends to remove remaining adapter sequences from reads. Reads were mapped to Bowtie2 (v 2.4.1)^50^ indexed human genome (GRCh38) using Bismark (v0.22.3) in non-directional and paired-end mode^51^. The methylation extraction module from Bismark was then used to produce coverage files for methylation analysis.

Coverage of annotated transposable elements was measured in scTEM-seq data using SeqMonk (v1.46.0)^52^. We excluded cells with coverage of less than 1000 annotated TE sites (or 500 for HL60 cells) using Repbase annotations. Methylation levels were calculated from .cov files using the mean of all CpG sites covered (Figs. 1C,D, 2B, 3 and Supplementary Figs. S7 and S8).

### Data processing and analysis (PBAT)

PBAT libraries were trimmed using Trim Galore to remove 9 bp from the 5’ end of all reads. Reads were mapped using Bismark in non-directional and paired-end mode. Unmapped reads were re-aligned in single-end mode to account for chimeric reads seen in PBAT libraries^53^. After producing coverage files with the Bismark methylation extraction module, paired and single end alignments for each sample were merged into a single file using the cat (concatenate) command. Downstream analysis was performed using SeqMonk. Genome wide cytosine methylation levels was averaged over 3000 bp tiles. SINE Alu methylation levels were measured over annotated Alu sites using Repbase annotations.

### Data processing and analysis (scRNA-seq)

scRNA-seq data was trimmed using Trim Galore, with default setting in paired-end mode. Hisat2^54^ (v2.1.0) and Samtools^55^ (v1.10) were used to convert, map and align unique and ambiguous reads to the human reference genome build GRCh38 from raw fastq reads into bam format. TEtranscripts^56^ was used to obtain raw gene and transposable element counts from the unique and ambiguously aligned reads using the GTF files for 1) TEs (http://labshare.cshl.edu/shares/mhammelllab/www-data/TEtranscripts/TE_GTF/) and 2) genes (https://asia.ensembl.org/info/data/index.html; release 101 from the FTP server) in GRCh38 ensembl format. TEtranscripts was run in a Conda^57^ environment setup with Python (v3.7.7)^58^, Pysam (v0.16.0.1)^59^, R-base (v4.0.3) and Bioconductor-Deseq2 (v1.28.0)^60^.

Correlation of gene and TE expression to DNA methylation (Fig. 2, Supplementary Fig. S6) was performed using R^61^. Transcripts with at least 2 reads in 10 cells were included in analysis. Read counts for scRNA-seq data were normalised per million reads for each sample and log transformed. Cor.test function using Pearson’s method was used to correlate gene and TE transcript counts with DNA methylation levels. P-values for significance of correlation were adjusted for false discovery rates using the p.adjust function and fdr method. Gene ontology was performed on genes of interest from correlation analysis using Panther^62^ statistical overrepresentation analysis. Panther’s GO biological process complete dataset was used for gene annotation, and expressed genes (at least 10 reads in 2 cells) were used as a reference list for the statistical overrepresentation analysis. Correlation, boxplots, and gene ontology results were plotted using ggplot2 (v3.3.5)^63^.

Differential expression analysis was performed in R using DESeq2 (v1.32.0)^60^ on genes and TEs at the family level (sum of TE element counts) on cells passing initial library QC and excluding features (genes and TEs) with less than 5 reads in at least 3 cells. Default parameters were used in DESeq2 with the significance threshold set at p adjusted < 0.05. Heatmapping was performed on all TE elements belonging to the ‘significantly differentially expressed’ TE families. Genes and TE counts (at the element level) were normalised by variance stability transformation (vst) (DESeq2), and the subset of TE elements were extracted, mean centred, and pheatmap (v1.0.12) ^64^ was used to produce the heatmaps with clustering by Euclidean distance on both rows (TEs) and columns (cells), with additional labels for treatment, corresponding global methylation levels and the TE ‘family’ each ‘element’ belongs.

## Supplementary Information

Supplementary Information.

Supplementary Tables.

## Data Availability

Sequencing data has been deposited in GEO database under accession number GSE171029.
