# Supplementary material for: scTEM-seq: Single-cell analysis of transposable element methylation to link global epigenetic heterogeneity with transcriptional programs
Source: Sci Rep. 2022 Apr 6;12:5776. doi: 10.1038/s41598-022-09765-x (PMC8986802; doi:10.1038/s41598-022-09765-x)
Supplement: Supplementary file 1 — Supplementary Information. [file 41598_2022_9765_MOESM1_ESM.docx]

**SUPPLEMENTARY METHODS**

**Data processing and analysis (scBS-seq)**

Analysis was performed on data published by Bian et al. (2018)^1^, downloaded from European Nucleotide Archive (ENA) (study accession PRJNA382695). First, raw reads were trimmed in single-end mode using Trim Galore (v0.6.5) with 9bp removed from the 5’ end of each read. Secondly, trimmed reads were mapped using Bismark (v0.22.3) in non-directional mode to a bowtie2 indexed human genome (GRCh38). Mapped reads were then deduplicated by Bismark with default settings. The methylation extraction module from Bismark was then performed on the deduplicated reads, with the resulting coverage files for read pairs merged into a single file.

Coverage files were then used to assess whether TEs can estimate global DNA methylation levels in single-cell data (Supplementary Figure S1). Methylation averages were first calculated using all cytosines covered in a library (genome methylation). Next, the GenomicRanges^2^ package was used to identify cytosines within annotated SINE Alu and LINE-1 elements (obtained from Repbase^3^), before the average methylation was calculated for these cytosines (SINE Alu and LINE-1 methylation).

**TE family analysis**

For analysis of TE family representation (Supplementary Figure S4), aligned scTEM-seq reads from .bam files were compared to annotated TEs in the reference genome (hg38) obtained from RepeatMasker^4^. The GenomicRanges^2^ package was used to count annotated TEs covered by at least 50bp of a scTEM-seq read. Super-families were identified using the “matching_class” field from the RepeatMasker annotation file, and “repeat_id” was used to identify SINE families. The base-R (v4.0.3)^5^ ‘heatmap’ function was used for visualisation.

**scTEM-seq bootstrapping analysis**

We used bootstrap analysis involving random sampling with replacement to estimate accuracy of scTEM-seq methylation calls (Supplementary figure S5C). Bootstrapping analysis was performed on bam files after mapping for 4 KG1a cells (2 from each treatment group) selected for high read counts. Samtools^6^ (v1.10) was used to subsample bam files at decreasing increments from 95% to 0.25% of the total file and create new subsampled bam files for analysis. Subsampled bam files were processed as standard scTEM-seq data and methylation estimates calculated. The process was repeated 50 times at each sampling increment to develop a distribution of methylation estimates. Results were visualised using ggplot2 (v3.3.5)^7^.

**scBS-seq library preparation, sequencing and analysis**

To compare scTEM-seq to an established, genome-wide, DNA methylation analysis method (Supplementary Figure S5A), we prepared scBS-seq libraries as described previously^8^ but with minor modifications. The ratio of AMPureXP beads used to purify first and second strand synthesis products was reduced from 0.75:1 to 0.65:1, and the 6NR adaptor 2 oligo used during second strand synthesis was modified (5’-CAGACGTGTGCTCTTCCGATCTNNNNNN-3’) to be compatible with NEBNext dual index oligos that were used for library amplification instead of PE1.0 and iTag index primers. Multi-plexed scBS-seq libraries were sequenced using the Illumina NextSeq platform, achieving an average 2.36x10^6^ raw read pairs per sample. Data processing was performed as described previously^8^, and the GenomicRanges^2^ package was used to identify cytosines covered by annotated SINE Alu elements (obtained from Repbase^3^). Average DNA methylation values were then calculated for all CpGs and SINE Alu CpGs in each CpG coverage file.

**SUPPLEMENTARY FIGURES**

**Supplementary Figure S1: Transposable element DNA methylation accurately reflects global levels in single-cell data.**

Single-cell data from a colorectal cancer patient (CRC01) were obtained from Bian *et al.* 2018 (GSE97693)^1,9^. Bisulphite sequencing libraries were processed as described in methods and DNA methylation averages were calculated for all cytosines, or those within TE annotations. A) DNA methylation in TEs (SINE Alu and LINE-1) correlates with genome wide levels. R^2^ and p values calculated using Pearson’s correlation. B) Genetic sub-lineages were defined by copy number analysis in Bian *et al.* 2018. DNA methylation in TEs was sufficient to detect the global hypomethylation observed in sub-lineages B0 and B1. All plots are coloured by sampling regions. LN = lymph node metastasis, ML = liver metastasis, MP = post-treatment liver metastasis, NC = adjacent normal colon, PT = primary tumour.

**Supplementary Figure S2: Amplicon and primer design for scTEM-seq.**

A) Comparison of unique TE sites measured against the number of uniquely mapped reads. Using scTEM-seq, greater coverage of SINE Alu was achieved per sequencing reads than LINE-1. B) Location of SINE Alu amplicon in relation to the consensus sequence. C) scTEM-seq library preparation showing amplification and incorporation of index and adapter sequences. Sequence-specific amplification of SINE Alu elements is first performed using oligos carrying partial adaptor sequences. A spacer of 0-5 random nucleotides is included to artificially increase library complexity by phasing amplicon reads. Internal dual index sequences are also included so that 96 single cell libraries can be pooled before a second amplification is used to introduce full sequencing adaptors. The adaptors also carry dual index sequences, so that up to 192 pools of 96 single cells can be sequenced in parallel.

**Supplementary Figure S3: Quality control of scTEM-seq libraries.**

A) Amplicon yield and C) mapping efficiency in DAC treated and untreated KG1a cells and negative controls. B) Amplicon yield and D) mapping efficiency in untreated AML01 patient cells and negative controls. Boxplots are in the style of Tukey, displaying Q1, Q3 and median.

**Supplementary Figure S4: Coverage of TEs in scTEM-seq libraries.**

KG1a scTEM-seq libraries that passed quality control were assessed for coverage of different A) TE super-families, and B) SINE families. Aligned reads for each library were compared to annotated TEs in the reference genome (GRCh38/hg38) obtained from Repbase. For an individual TE annotation to be counted as covered in a library, a minimum overlap of 50bp with a scTEM-seq read was required. The total number of TEs covered in each library was then calculated for A) TE super-families and B) SINE families. In the heatmaps, TEs (rows) are arranged by their total count in all scTEM-seq libraries, and cells (columns) are arranged by their total count of all TEs. The total number of TE annotations in the reference genome (hg38) is also indicated for A) TE super-families and B) SINE families (colour scale at left).

**Supplementary Figure S5: Validation of scTEM-seq data.**

A) DNA methylation heterogeneity observed in scTEM-seq data was compared to scBS-seq libraries prepared from decitabine (DAC) treated KG1a cells. DNA methylation estimates from scBS-seq libraries were calculated using either all CpGs, or only CpGs in annotated SINE Alu elements. scTEM-seq and scBS-seq libraries had similar distributions of DNA methylation levels, with no significant difference in variance (two-sided F-test) or means (two-sided Welch t-test assuming equal variance) observed. Boxplots are in the style of Tukey, displaying Q1, Q3 and median. B) DNA methylation levels as measured by scTEM-seq in HL60 cells with and without DAC treatment (100nM, 72h). C) KG1a cells with high read counts were selected for bootstrap subsampling analysis to assess stability and accuracy of methylation estimates using scTEM-seq. Subsampling was performed using Samtools on mapped reads at decreasing increments to 0.25% of reads. 50 subsamples were produced at each increment and mean methylation was calculated to produce a range of estimates. Cells G11 and H12 are untreated and cells D11 and B10 are DAC treated.

**Supplementary Figure S6: Transcription of TEs in single-cell data.**

A) Comparison of the average normalised expression values (log_10_(RPM)) for genes (grey dots) and TEs (red dots) in DAC treated vs untreated KG1a cells. An intersect line (y=x) is plotted with any gene or TE above the line representing a relative increase in average expression in DAC treated compared to untreated. B) Volcano plots showing Pearson’s correlation coefficients between DNA methylation levels and TE expression for KG1a, AML01 and HL60 datasets. The dashed line in the HL60 plot indicates the significance threshold. FDR = False Discovery Rate.

**Supplementary Figure S7: Heatmap of TE expression in AML01 blasts.**

The heatmap shows the relative expression of TE elements from patient (AML01) cells, following normalisation by variance stabilisation transformation (vst) (DESeq2) and mean centering, with both rows (TEs) and columns (cells) clustered by Euclidean distance. Global DNA methylation percentages for each cell are indicated (green scale at top) and selected TE families are highlighted (left). The TEs used in this heatmap belonged to the TE families identified in the differential expression analysis of untreated vs DAC treated KG1a cell (Fig. 3).

**Supplementary Figure S8: Heatmap of TE expression in HL60 cells.**

TE families with altered expression between untreated (red) and DAC treated (blue) KG1a cells were identified by differential expression analysis using DEseq2. The heatmap shows the relative expression of all TEs from significant families (adjusted p<0.05) following normalisation by variance stabilisation transformation (vst) (DESeq2) and mean centering. Both rows (TEs) and columns (cells) clustered by Euclidean distance. Global DNA methylation percentages for each cell are indicated (green scale at top) and selected TE families are highlighted (left). In total, 12 TE families reached the significance threshold (Family:Class; ERV1:LTR, centr:Satellite, MIR:SINE, hAT-Tip100:DNA, L2:LINE, ERVL:LTR, ERVL-MaLR:LTR, TcMar-Tigger:DNA, L1:LINE, Satellite:Satellite, hAT-Blackjack:DNA, RTE-X:LINE), corresponding to 701 TE elements. A sub-cluster of mostly DAC treated cells (right) have high expression of TEs.

**Supplementary Figure S9: DNA methylation of DAC treated cells separated by TE expression cluster.**

Methylation of DAC treated KG1a (A) and HL60 (B) cells from clusters identified in Figure 3 and Supplementary Figure S8. The blue boxplots represent DAC-treated cells with increased expression of TEs. Boxplots are in the style of Tukey, displaying Q1, Q3 and median.

**SUPPLEMENTARY TABLES**

**Supplementary Table S1: scTEMseq primer sequences.**

**Supplementary Table S2: scTEMseq primer pairs (96 well plate layout).**

**Supplementary Table S3: Quality control of scTEM-seq libraries prepared from KG1a AML cells.**

**Supplementary Table S4: Quality control of scTEM-seq libraries prepared from primary AML cells.**

**Supplementary Table S5: Quality control of scRNA-seq libraries prepared from KG1a AML cells.**

**Supplementary Table S6: Quality control of scRNA-seq libraries prepared from primary AML cells.**

**Supplementary Table S7: Comparison of scTEM-seq to selected methods for genome-wide DNA methylation analysis.**

| **Method** | **scTEM-seq** | **scBS-seq** | **snmC-seq2** | **sci-MET** |
| --- | --- | --- | --- | --- |
| Reference | This Paper | Angermueller *et al* (2016)^10^ | Luo *et al* (2018)^11^ | Mulqueen *et al* (2018)^12^ |
| Library Preparation Cost (USD / Sample) | ~ $10 | ~ $20 | ~ $10 | < $1 |
| Library Preparation Time (Days) | 2 | 3 | 2 | 3 |
| Library Throughput (Samples / Batch) | 96 (initial steps) | 96 (all steps) | 96 (initial steps) | 700 |
| Sequencing Demand (Raw Reads / Library) | 25,000 | 11,000,000 | 5,000,000 | 750,000 |
| Mapping Efficiency (Reads with Unique Alignment) | 75% | 15% | 65% | 55% |
| Information Obtained  (CpGs / Library) | 20,000 | 2,500,000 | 2,000,000 | 165,000 |
| Parallel Transcriptomics | Yes | Yes | Theoretically possible | Not compatible |

**SUPPLEMENTARY REFERENCES**

1	Bian, S. *et al.* Single-cell multiomics sequencing and analyses of human colorectal cancer. *Science* **362**, 1060-1063, doi:10.1126/science.aao3791 (2018).

2	Lawrence, M. *et al.* Software for Computing and Annotating Genomic Ranges. *PLOS Computational Biology* **9**, e1003118, doi:10.1371/journal.pcbi.1003118 (2013).

3	Bao, W., Kojima, K. K. & Kohany, O. Repbase Update, a database of repetitive elements in eukaryotic genomes. *Mobile DNA* **6**, 11-11, doi:10.1186/s13100-015-0041-9 (2015).

4	Smit, AFA, Hubley, R & Green, P. RepeatMasker Open-4.0 (2013-2015).

5	R: A Language and Environment for Statistical Computing (2020).

6	Li, H. *et al.* The Sequence Alignment/Map format and SAMtools. *Bioinformatics* **25**, 2078-2079, doi:10.1093/bioinformatics/btp352 (2009).

7	Wickham, H. *ggplot2: Elegant Graphics for Data Analysis*, <<https://ggplot2.tidyverse.org>> (2016).

8	Clark, S. J. *et al.* scNMT-seq enables joint profiling of chromatin accessibility DNA methylation and transcription in single cells. *Nature Communications* **9**, 781, doi:10.1038/s41467-018-03149-4 (2018).

9	Estey, E. H. Epigenetics in clinical practice: the examples of azacitidine and decitabine in myelodysplasia and acute myeloid leukemia. *Leukemia* **27**, 1803-1812, doi:10.1038/leu.2013.173 (2013).

10	Angermueller, C. *et al.* Parallel single-cell sequencing links transcriptional and epigenetic heterogeneity. *Nature methods* **13**, 229-232, doi:10.1038/nmeth.3728 (2016).

11	Luo, C. *et al.* Robust single-cell DNA methylome profiling with snmC-seq2. *Nature Communications* **9**, 3824, doi:10.1038/s41467-018-06355-2 (2018).

12	Mulqueen, R. M. *et al.* Highly scalable generation of DNA methylation profiles in single cells. *Nat Biotechnol* **36**, 428-431, doi:10.1038/nbt.4112 (2018).
